# Skeletal Muscles Do Not Undergo Apoptosis During Either Atrophy or Programmed Cell Death-Revisiting the Myonuclear Domain Hypothesis

**DOI:** 10.3389/fphys.2018.01887

**Published:** 2019-01-25

**Authors:** Lawrence M. Schwartz

**Affiliations:** Department of Biology, Morrill Science Center, University of Massachusetts, Amherst, MA, United States

**Keywords:** myonuclei, *Manduca sexta*, autophagy, sarcopenia, intersegmental muscle

## Abstract

Skeletal muscles are the largest cells in the body and are one of the few syncytial ones. There is a longstanding belief that a given nucleus controls a defined volume of cytoplasm, so when a muscle grows (hypertrophy) or shrinks (atrophy), the number of myonuclei change accordingly. This phenomenon is known as the “myonuclear domain hypothesis.” There is a general agreement that hypertrophy is accompanied by the addition of new nuclei from stem cells to help the muscles meet the enhanced synthetic demands of a larger cell. However, there is a considerable controversy regarding the fate of pre-existing nuclei during atrophy. Many researchers have reported that atrophy is accompanied by the dramatic loss of myonuclei *via* apoptosis. However, since there are many different non-muscle cell populations that reside within the tissue, these experiments cannot easily distinguish true myonuclei from those of neighboring mononuclear cells. Recently, two independent models, one from rodents and the other from insects, have demonstrated that nuclei are not lost from skeletal muscle fibers when they undergo either atrophy or programmed cell death. These and other data argue against the current interpretation of the myonuclear domain hypothesis and suggest that once a nucleus has been acquired by a muscle fiber it persists.

## Muscle Plasticity

Skeletal muscle is the largest tissue in the body. It is highly plastic and can undergo dramatic non-pathological swings in both mass and strength in response to a myriad of environmental influences. Following resistance exercise or exposure to anabolic steroids, muscles undergo hypertrophy and increase its mass, cross-sectional area, and strength (Aagaard, 2004; Schiaffino et al., 2013). These same parameters can decline precipitously when muscles initiate atrophy in response to any of the range of insults that include: starvation, denervation, immobilization, sepsis, cancer cachexia, cardiac failure, diabetes, renal failure, chronic obstructive pulmonary disorder, and acquired immunodeficiency syndrome (Schiaffino et al., 2013).

The consequences of atrophy have obvious implications for health. Muscle weakness is a major contributor to both mortality and morbidity and is associated with the increased risk of all causes of death (Metter et al., 2002). In fact, reducing muscle atrophy in cancer cachexia can significantly prolong life (Zhou et al., 2010). As well, many older individuals suffer from sarcopenia, a protracted muscle wasting disorder that typically begins after the age of 50 and involves a loss of about 1% of muscle mass per year (Woo, 2017). This means that by the age of 80, sarcopenic individuals have lost about 40% of their muscle mass, a key factor in falls, frailty, and nursing home admissions. Consequently, understanding the mechanisms and potential therapeutic responses to atrophy is of broad basic and clinical interest (Ali and Garcia, 2014; Cohen et al., 2015; Ziaaldini et al., 2017).

## Myonuclear Domain Hypothesis

Skeletal muscle is fairly unique in that the mature cells are syncytial and can contain hundreds of nuclei. This is a necessary adaptation given that individual muscle fibers can be enormous, achieving lengths of up to ~600 mm (23 in) (e.g. sartorius muscle; Yang et al., 1998) and volumes that can be more than 100,000 times greater than a typical mononucleated cell (Bruusgaard et al., 2003). The contributions from multiple nuclei are required to produce large amounts of mRNA needed to direct the prodigious protein synthesis required to generate and maintain the contractile apparatus (Nevalainen et al., 2013).

Both the plastic nature of muscle and its syncytial organization have given rise to a controversy that only recently appears to have been resolved—the “*myonuclear domain hypothesis*” (Qaisar and Larsson, 2014; Gundersen, 2016; Schwartz et al., 2016). This theory has its origins in the concept of “Wirkungssphäre” or “sphere of influence” proposed by Strassburger (1893), in which he argued that a nucleus can only support a discrete volume of cytoplasm, thus defining the upper limits to cell size. This principle was elaborated further by Gregory (2001) who argued that cellular deoxyribonucleic acid content and volume are tightly coupled. The syncytial nature of skeletal muscle helps these cells overcome this limitation. The myonuclear domain hypothesis dictates that in order to maintain the proper nuclear-to-cytoplasmic ratio, new nuclei are added during hypertrophy and lost with atrophy.

There is substantial data demonstrating that nuclear number does increase with muscle hypertrophy (Moss, 1968; Cabric and James, 1983; Egner et al., 2016), although some controversy remains (Murach et al., 2018). This process has been an area of intense investigation and some of the underlying molecular mechanisms that regulate the acquisition of new nuclei during hypertrophy have been defined recently (Guerci et al., 2012; Bentzinger et al., 2014; Ross et al., 2018). These supernumerary nuclei are acquired when lineage-restricted stem cells, typically satellite cells, fuse with muscle fibers and contribute their nuclei (Brack and Rando, 2012; Bachman et al., 2018). Satellite cells reside under the basement membrane and abut the sarcolemma (Katz, 1961; Mauro, 1961). They remain quiescent until stimulated by either anabolic steroids like testosterone or by focal injury following resistance exercise, at which point they reenter the cell cycle and proliferate (Joubert and Tobin, 1995; Abreu et al., 2017). Some of the daughter cells fuse with the muscle fiber and facilitate both repair and growth, while others arrest and reconstitute the satellite pool (Dumont et al., 2015; Goh and Millay, 2017). The remaining surplus cells undergo apoptosis (Schwartz, 2008).

The controversial aspect of the myonuclear domain hypothesis is the contention that myonuclei are lost during atrophy. There are many interventions that induce atrophy in animal models, including: immobilization, denervation, and sepsis (Fitts et al., 1986; Minnaard et al., 2005; O’Leary et al., 2012). In each case, there is a net loss of both muscle cross-sectional area (the primary assay) and the appearance of apoptotic cells within the tissue (McCall et al., 1998; Smith et al., 2000; Strasser et al., 2000; Alway et al., 2003; McClung et al., 2007; Andrianjafiniony et al., 2010; Guo et al., 2012; Palumbo et al., 2012; Barnes et al., 2015; Cheema et al., 2015; Li et al., 2016; Kletzien et al., 2018). In these studies, apoptosis is measured in a variety of methods, including caspase activation, mitochondrial EndoG release, or DNA fragmentation [which is detected anatomically with terminal deoxynucleotidyl transferase (TdT) dUTP Nick-End Labeling (TUNEL) staining or biochemically by visualizing DNA ladders *via* agarose gel electrophoresis]. These studies provide compelling data that apoptosis increases dramatically during the early phase of atrophy. For example, in a recent comprehensive study (Guo et al., 2012), Guo et al. subjected mice to 14 days of hindlimb suspension, a treatment that resulted in a ~69% reduction in muscle wet weight and an ~43.8% reduction in cross-sectional area relative to the unmanipulated contralateral muscle. The authors also quantified a number of apoptosis markers, including TUNEL staining, caspase-3 cleavage/activation, and the cleavage of poly(adenosine diphosphate ribose) polymerase (PARP), a protein involved in DNA repair. Each of these apoptosis measures increased significantly following hindlimb suspension. While the primary focus of this paper was to evaluate the positive impact of electrical stimulation on limiting atrophy following an insult, they and many other researchers interpret these kinds of data as providing strong support for the myonuclear domain hypothesis.

From a cell biological perspective, the presumptive loss of nuclei within a syncytial tissue like skeletal muscle presents a major practical problem. How can an individual nucleus become so compromised that its genome rapidly condenses and fragments while its neighbors persist and help maintain the viability of the muscle fiber? Given that apoptosis is typically mediated by the activation of the class of cysteine proteases known as caspases, it is not clear what mechanism might serve to restrict the activity of a diffusible protease within a common cytoplasm.

This question has been addressed indirectly in another syncytial cell type, the human syncytiotrophoblast, a tissue that surrounds the placenta and contains about 5 × 10^10^ nuclei (Mayhew et al., 1999). When apoptosis is induced in the syncytiotrophoblast, it propagates as a wave at a rate of about 5 microns per minute until the entire tissue is involved (Longtine et al., 2012). Consequently, there are no “privileged” regions within the syncytial cytoplasm and all of the nuclei are ultimately destroyed.

One of the key challenges with analyzing apoptosis in skeletal muscle is that it is a very heterogeneous tissue, where approximately half of its nuclei reside outside muscle fibers (Schmalbruch and Hellhammer, 1977). These mononucleated cells include satellite cells, endothelial cells, fibroblasts, pericytes, and macrophages (Tedesco et al., 2010). Consequently, it is very difficult to determine which side of the sarcolemma, a nucleus resides, and thus if it is a true myonucleus.

## *In Vivo* Time-Lapse Imaging of Labeled Mouse Muscle Fibers

Despite the large number of papers demonstrating apoptosis during muscle atrophy, several authors have questioned these results (Wada et al., 2002; Zhong et al., 2005; Aravamudan et al., 2006; Gundersen and Bruusgaard, 2008; Duddy et al., 2011; Qaisar and Larsson, 2014). For example, using isolated muscle fibers *in vitro*, Duddy et al. noted that while muscle fiber volume decreased over time, reflective of an atrophic process, the number of myonuclei appeared to be stable (Duddy et al., 2011).

**Figure 1:**
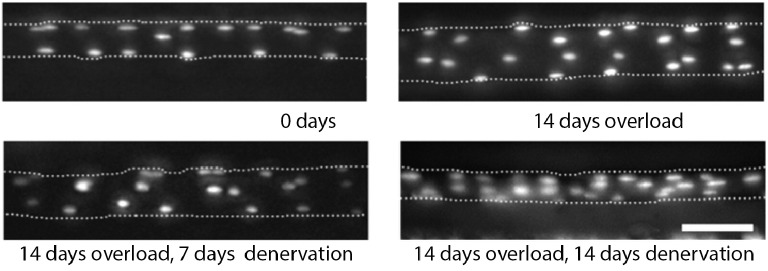
Myonuclei are acquired during hypertrophy but not lost during atrophy in mouse. Micrographs of same EDL muscle fiber over time following the induction of hypertrophy (top row) and the subsequent induction of atrophy (bottom row). Fluorescently labeled oligonucleotides were used to visualize the nuclei *in vivo*. The dotted lines represent the sarcolemma. Scale bar = 50 μm. (Adapted from Bruusgaard et al., 2010. Used by permission of the Proceedings of the National Academy of Sciences.)

In a series of elegant experiments, the Gundersen lab injected individual extensor digitorum longus (EDL) or soleus muscles muscle fibers with dyes that independently labeled the nuclei and cytoplasm in anesthetized mice (Bruusgaard and Gundersen, 2008; Bruusgaard et al., 2010). This allowed them to identify each nucleus within individual muscle fibers *in vivo* over time and then evaluate its fate. For example, EDL muscles were induced to hypertrophy by the ablation of their major synergists (Bruusgaard et al., 2010). Between days 6 and 11, the number of myonuclei increased by about 54% and between days 9 and 14 there was a 35% increase in cross-sectional area (Figure 1). These data are consistent with the hypothesis that muscles acquire supernumerary nuclei in advance of the major growth of the fiber during hypertrophy.

They then examined the fate of these uniquely identifiable myonuclei in the same muscle fibers when they were induced to undergo atrophy. Denervation led to an approximately 50% reduction in muscle fiber volume, but no loss of myonuclei (Figure 1; Bruusgaard et al., 2010). The failure to observe nuclear loss was not due to the atrophic stimulus employed since they obtained the same results when the muscles were induced to atrophy in response to tetrodotoxin-induced nerve blockade, hindlimb suspension, cancer cachexia, or detraining (Bruusgaard and Gundersen, 2008; Bruusgaard et al., 2010; Winje et al., 2018a). In fact, during the course of their studies, they examined more than 200,000 individual myonuclei in atrophic muscles and observed only 4 TUNEL-positive (apoptotic) nuclei, which represents a loss of only ~0.002% of the nuclei (Bruusgaard et al., 2012). These data strongly support the hypothesis that skeletal muscle atrophy is not accompanied by myonuclear death.

## Insect Muscle as a Model

As powerful as these studies are, there are some limitations inherent with the use of mammalian models. First, they invariably rely on experimental interventions such as denervation to induce muscle atrophy. Second, the contralateral muscle is typically used as an internal control even though it too is exposed to some of the same environmental signals, such as stress hormones (Bonaldo and Sandri, 2013). Lastly, skeletal muscles can also undergo programmed cell death (PCD) during development, and these models rarely address the role of apoptosis in these cells.

**Figure 2:**
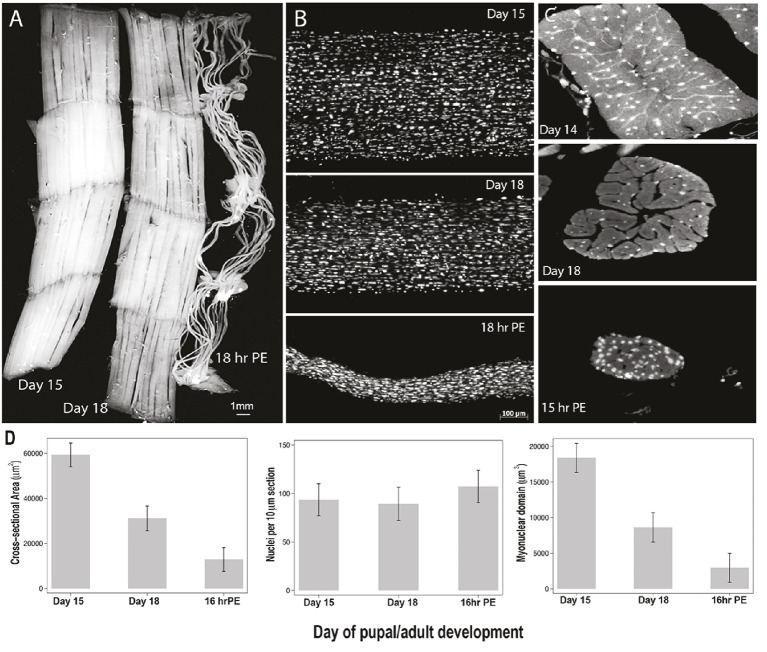
Retention of myonuclei during both atrophy and death of the intersegmental muscles (ISMs) from the moth *Manduca sexta*. **(A)** ISMs from three stages of development: homeostatic (days 15 of pupal-adult development; left); atrophic (day 18; middle); and dying (18 h post-eclosion; right). Scale bar equals ~1 mm (adapted from Schwartz et al., 2016). **(B)** ISMs from these same developmental stages were cleared and stained with the nuclear dye DAPI and visualized *via* confocal microscopy (adapted from Schwartz et al., 2016). **(C)** ISM fiber sections (10 μm) were stained with the nuclear dye DAPI. Note the dramatic loss of muscle protein (light gray area) during atrophy and death, but the retention of nuclei at all stages (adapted from Schwartz et al., 2016). **(D)** Quantification of ISM fiber volume (left), nuclear number (middle), and myonuclear domain size (right) during homeostasis, atrophy, and death. (Mean ± standard error.) (Adapted from Schwartz et al., 2016).

An alternative model that does not suffer from any of these limitations, and allows the study of muscle nuclei during both atrophy and naturally occurring PCD, is the intersegmental muscle (ISM) from the tobacco hawkmoth *Manduca sexta*. The ISMs are composed of sheets of giant muscle fibers, where each cell is about 5 mm long and up to 1 mm in diameter (Figure 2A). The ISMs attach to the segmental boundaries within the abdomen and generate both the crawling behavior of the larvae and the eclosion (emergence) behavior of the adult moth when it escapes from the pupal cuticle at the end of metamorphosis.

On day 15 of the normal 18 days of pupal-adult development, the ISMs initiate a hormonally triggered program of atrophy that results in a 40% loss of mass by the time of eclosion 3 days later (Figure 2A; Schwartz and Truman, 1983). This dramatic loss of muscle mass is equivalent to that seen in 80-year-old individuals with sarcopenia, but takes place over 3 days rather than 30 years. While the ISMs lose mass, they retain normal physiological properties such as resting potential and force/cross-sectional area (Schwartz and Ruff, 2002). The ISMs are used to generate the eclosion behavior at the end of day 18 and then initiate PCD, both of which are triggered by a peptide hormone (Schwartz and Truman, 1982, 1983). [It should be noted that the term PCD was originally coined to describe the death of the ISMs, so this model represents the classic cell death system (Lockshin and Williams, 1965)].

In contrast to mammalian muscles, the ISMs do not contain capillaries, satellite cells, endothelial cells, or pericytes, meaning that virtually all of the nuclei in the tissue reside within the muscle fibers themselves (Beaulaton and Lockshin, 1977). We used two independent methods to monitor nuclear fate during both atrophy and death in this model (Schwartz et al., 2016). The first was a standard anatomical approach. When the ISMs undergo atrophy and death, there are dramatic reductions in both the volume and cross-sectional area of the fibers, but grossly, nuclear number was unchanged (Figure 2B). Using sectioned tissues for quantitative studies, we found that there was a ~49% reduction in fiber cross-sectional area during atrophy and a further 30% decline during the early stages of death (Figures 2C,D; Schwartz et al., 2016). However, the number of nuclei did not change during this same period meaning that there was functionally an 84% reduction in the myonuclear domain.

The second method we employed was to measure the DNA content of individual muscle fibers from animals on day 13, when the muscles are homeostatic, until 18 h post-eclosion, when the muscles are highly degenerate (Schwartz et al., 2016). We quantified the DNA content of 420 individual cells and found that there was no significant loss during development. These biochemical analyses agree well with the anatomical data and suggest that the relative concentration of both nuclei and genomic DNA functionally increased as the muscles atrophied and died. Consequently, data from both mice and moths suggests that muscle nuclei do not undergo apoptosis during atrophy (or PCD) and leads to the conclusion that the myonuclear domain hypothesis should be rejected.

## Conclusions

These observations have a number of implications for both understanding the basic biology of muscle and for developing potential therapeutic interventions. While there is substantial data reporting the presence of apoptotic nuclei within the tissue following atrophic insults, recent data suggests that these are not true myonuclei, but rather, condemned mononuclear cells that reside outside the muscle fiber. The primary reason for this discrepancy is that the traditional tools used for detecting apoptosis lack the resolution required to adequately determine which side of the sarcolemma a dying cell resides. Methods that label myonuclei specifically, either *via in vivo* injections of fluorescent dyes (Bruusgaard and Gundersen, 2008) or *via* genetic manipulations to express marker proteins (Duddy et al., 2011), reveal few if any apoptotic nuclei within the labeled fibers. The recent demonstration that antibodies directed against Protein Pericentriolar Material 1 (PCM1) label only authentic myonuclei offers a promising tool that can be applied to the traditional histological materials that are routinely analyzed in the field (Winje et al., 2018b).

The failure of skeletal muscles to employ apoptosis as a mechanism for eliminating myonuclei is not surprising. It appears that mature skeletal muscle is largely precluded from initiating apoptosis in that it upregulates the expression of potent survival proteins like X-linked inhibitor of apoptosis protein (XIAP) (Smith et al., 2009) and apoptosis repressor with caspase recruitment domain (ARC) (Xiao et al., 2011), while simultaneously expressing low levels of apoptosis mediators like apoptotic protease activating factor 1 (Apaf-1) (Burgess et al., 1999). This makes sense teleologically since muscles are subject to extreme perturbations, including disruption of the sarcolemma following intense exercise, so it would be catastrophic if they triggered cell death rather than initiated tissue repair and possibly hypertrophy. Like other terminally differentiated cells, muscles tend to employ non-apoptotic mechanisms, most notably autophagic PCD (also known as Type II degeneration) (Clarke, 1990; Schwartz et al., 1993; Kole et al., 2013; Ginet et al., 2014) when they die during development.

Even if there was a mechanism by which muscles could selectively target individual nuclei, it is not clear that this would be beneficial. In fact, the retention of “surplus” nuclei during atrophy confers a distinct advantage for the individual since skeletal muscles frequently undergo cycles of atrophy and hypertrophy in response to environmental conditions such as food availability. The ability to recover quickly by utilizing pre-existing myonuclei may serve an important role in adaptation (Jackson et al., 2012) and help explain the phenomenon of “muscle memory” (Staron et al., 1991; Gundersen, 2016). It is well documented in the field of exercise physiology that it is far easier to reacquire a certain level of muscle fitness through exercise than it was to achieve it the first place, even if there has been a long intervening period of detraining. In other word, the phrase “use it or lose it” is might be more accurately articulated as “use it or lose it, until you work at it again.” This has been demonstrated directly by another experiment from the Gundersen lab that demonstrated that once a muscle has acquired new nuclei, it retains them long after the hypertrophic stimulus is removed. They induced muscle hypertrophy in female mice by treating them for 2 weeks with testosterone and then examined the muscles 3 weeks after steroid withdrawal (Egner et al., 2013). Muscle volume had returned to baseline but the newly acquired nuclei persisted even 3 months later. When the muscles were subjected to overloading to reinitiate hypertrophy, the steroid-treated ones rapidly underwent a 36% increase in fiber volume while control muscles only grew by 6%. These data suggest that the “surplus” nuclei could be mobilized rapidly to facilitate retraining.

These observations have potential implications for public health policy. It has been shown that muscle growth, physiological function, and regenerative capacity all decline with age, largely due to reduced satellite cell proliferation (Blau et al., 2015). Consequently, exercise during adolescence, when muscle growth is enhanced by hormones, nutrition and a robust satellite pool, might functionally serve to allow individuals to “bank” myonuclei that could be drawn upon later in life to slow the effects of aging and possibly forestall sarcopenia.

In addition, these data have implications in the area of competitive sports. The use of anabolic steroids is a potent stimulus for muscle hypertrophy and the addition of new myonuclei (Egner et al., 2013). Since these nuclei persist long after the steroid use ends, athletes likely derive the benefits of illegal drug use without the risk of detection.

In summary, while the addition of new nuclei with muscle growth is largely accepted, the apoptotic loss of nuclei with atrophy cannot be supported, suggesting that strict interpretation of the myonuclear domain hypothesis cannot be supported. Instead, it appears that once acquired, myonuclei persist even when a muscle becomes atrophic or initiates cell death.

## Author Contributions

The author confirms being the sole contributor of this work and has approved it for publication.

### Conflict of Interest Statement

The author declares that the research was conducted in the absence of any commercial or financial relationships that could be construed as a potential conflict of interest.
